# Genotoxic Effect of Chronic Exposure to DDT on Lymphocytes, Oral Mucosa and Breast Cells of Female Rats

**DOI:** 10.3390/ijerph8020540

**Published:** 2011-02-18

**Authors:** Alejandro Canales-Aguirre, Eduardo Padilla-Camberos, Ulises Gómez-Pinedo, Hugo Salado-Ponce, Alfredo Feria-Velasco, Ruth De Celis

**Affiliations:** 1 Unit of Medical and Pharmaceutical Biotechnology, Center for Research and Assistance in Technology and Design of Jalisco, A.C. (CIATEJ), Normalistas 800, Guadalajara, 44270, Mexico; E-Mails: acanales@ciatej.net.mx (A.C.-A.); epadilla@ciatej.net.mx (E.P.-C.); hsalado@hotmail.com (H.S.-P.); 2 Laboratory of Regenerative Medicine, Neuroscience Institute, Hospital Clinico San Carlos, Madrid, 28040, Spain; E-Mail: ulisesalfonso.gomez@salud.madrid.org; 3 Biological Sciences Department, Biological and Agricultural Sciences Center, University of Guadalajara, Carr. a Nogales Km 15.5, Zapopan, 45110, Mexico; E-Mail: aferia@cucba.udg.mx; 4 BiosMedica Cancer Research Institute, Alcatraces 72, Jardin Real, Zapopan, 45136, Mexico; 5 Environmental Immunology Laboratory, Western Biomedical Research Center of the Mexican, Institute for Social Security, Sierra Mojada 800, Colonia Independencia, S.L., Guadalajara, 44340, Mexico

**Keywords:** genetic damage, xenohormones, micronuclei (MN), comet assay, lipid peroxidation

## Abstract

The genotoxicity of some environmental contaminants may affect human health directly by damaging genetic material and thus plays an important role in cancer development. Xenoestrogens are one kind of environmental pollutants that may alter hormonal routes or directly affect DNA. The number of available biomarkers used to assess genetic risk and cancer is very extensive. The present study evaluated genotoxicity produced by the pesticide DDT on systemic and mammary gland cells obtained from adult female Wistar rats. Oral mucosa cells micronuclei were assessed; the comet assay in peripheral blood-isolated lymphocytes and mammary epithelial cells was also carried out. Additionally, oxidative stress was studied in mammary tissue through a lipid peroxidation assay. Our data showed an increase in lipid peroxidation, product of an increase in free oxygen radical levels, which leads to an oxidative stress status. Our results suggest that DDT is genotoxic, not only for lymphocytes but also to mammary epithelial cells.

## 1. Introduction

There has been an increasing concern among the general and medical community in relation to the potential environmental hazard that estrogens present to health in general, although the mechanisms by which they affect homeostasis are still barely known [1–3]. It has been known for several years that exposure to some xenohormones increases the production of reactive oxygen species, which in turn could inflict structural damage to cell DNA of target organs, as well as to DNA from other systemic cells [4,5].

The genotoxic action of contaminating agents affects human health directly, damaging the genetic material, which is considered to play an important role in oncogenesis [6,7]. Of all cancer types, only about 10% is considered to be provoked by endogenous reasons (genetic and hormonal); the rest can be attributed to external environmental factors acting in conjunction with endogenous factors, where individual susceptibility is also important [8,9].

Genetic monitoring of human populations exposed to environmental mutagenic carcinogens can be performed through various biomarkers [10–12]. There is an extended availability of biomarkers for cancer and genetic risk evaluation, and their efficiency in bio-monitoring is established by the well known paradigm of environmentally induced cancer, which represents the final point of the human-genotoxics interaction spectrum. The micronuclei (MN) index in bone marrow and the peripheral erythrocyte count are a couple of the most accepted *in vivo* cytogenetic bioassays in the field of genetic toxicology [13]. This test can also be used in those tissues where exfoliated cells have been obtained [14,15]. On the other hand, the practice of the single cell electrophoresis test (comet assay) has obtained an increasing acceptance in the genetic toxicology field [16,17]; some of its advantages are: damage detection at an individual cell level, high sensitivity (mainly under alkaline conditions), as well as the possibility to use enzymes or antibodies to detect specific types of damage [17,18]. Oxidative stress is one of the best known causes of cellular damage, mostly due to the formation of free radicals that damage cell DNA [19]; in recent years malondialdehyde (MDA) concentration has been used as an oxidative stress biomarker [20].

Several studies have been performed in order to estimate the genotoxic and carcinogenic potential of environmental toxic contaminants [2], but these studies have yet to produce enough data to better understand the mechanisms involved in carcinogenesis. Although the 1,1,1-trichloro-2,2-bis-(chlorophenyl)-ethane (DDT), was a widely used insecticide during the malaria control program in Mexico and its use has been banned since 1999 [21,22], human exposure has continued as a result of its environmental persistence as well as from contact through other new sources.

It is known that DDT and dichlorodiphenyldichloroethylene (DDE), one of its metabolites in particular, are considered xenoestrogens [3,23], and have been associated to breast cancer etiopathogeny [24,25]. However, DDT-induced health effects are still in debate; thus, studies are needed in order to learn more about the toxicity of this insecticide. The aim of the present work is to evaluate the genotoxic effect of DDT exposure in oral mucosa cells, lymphocytes and mammary gland epithelial cells in adult female rats.

## 2. Experimental Section

### 2.1. Laboratory Animals

In the study we used thirty 40-day-old female Wistar rats, weighing between 100 and 150 g, which were divided into three groups. Animals were maintained throughout the experiments in a 12-h light: 12-h dark cycle (light-on at 7:00 h) and kept at 22 ± 1 °C with 50% relative environmental humidity. Purina Chow™ for rodents and water were provided *ad libitum*. All the experiments were carried out in accordance with the Guide for the Care and Use of Laboratory Animals as established by the National Institutes of Health (USA).

### 2.2. Exposure Assays

An exposed group was composed of 10 rats under exposure in chamber especially designed for this study to the environmental xenoestrogen DDT (CAS RN 50-29-3, reagent grade, 98% purity, Aldrich Chemical Co. Inc., Milwaukee, WI, USA), by means of inhalation to an approximate concentration of 7 mg/m^3^ (concentration considered to be a saturated atmosphere for enclosed areas by WHO reports, [26,27]), during 8 h per day, 6 days a week, for a period of five months,. A vehicle control group composed of 10 rats was kept under exposure to absolute ethanol IQ (CAS RN 64-17-5, Kem, Leon, Gto. Mex.) which was used as vehicle for DDT; in a corresponding volume to that used for the exposed group. A third group composed of 10 rats exposed to atmospheric air was used as the intact control.

### 2.3. Obtaining Samples

After the five-month treatment period, the animals were anesthetized by sodium pentobarbital (50 mg/kg) intraperitoneal injection. Mammary tissue was obtained through biopsy in order to perform the comet assay and the lipid peroxidation bioassay. For the comet assay, the tissue was macerated on a mortar adding a diluted trypsin solution in order to separate the epithelial cells. For MN quantification, surface epithelial cells from oral mucosa were obtained by scraping. After this, 4 mL of blood were obtained through cardiac puncture and kept at 4 °C in heparinized vacutainer tubes. Lymphocytes were obtained by centrifugating the blood in a Ficoll gradient and washed three times in a phosphate buffered-saline solution. Once lymphocytes were isolated, DNA damage was assessed by single cell electrophoresis.

### 2.4. Micronuclei in Oral Mucosa Cells

Smears of oral mucosa were fixed with cyto-spray and stained with the Papanicolaou technique [28]. All slides were observed under a light microscope equipped with CCD digital camera and computer software for image analysis (Q-Win Pro Leica Image Analyzer, Germany). For determination of the MN index, at least 500 epithelial cells were scored quantifying those with MN.

### 2.5. Single Cell Electrophoresis (Comet Assay)

The assay was performed on isolated lymphocytes or epithelial mammary cells according to Sigh *et al.* [29], with some modifications. Glass slides were prepared with three layers: (1) 0.65% agarose; (2) a cell suspension and 0.6% low melting point agarose mixture; (3) 0.6% low melting point agarose. These three layers were solidified in sequence at 4 °C, and subjected afterwards to a lysis step (1-hour 4 °C incubation in 1% *N*-laurylsarcosin, 2.5 mol/L NaCl, 100/L Na_2_EDTA, 1% Triton X-100 and 10% dimethylsulfoxide) to eliminate the cellular and nuclear membranes. After completion of the lysis step, the slides were placed for 45 min in the dark, in an ice-cold electrophoresis chamber containing alkaline electrophoresis buffer (300 mmol/L NaOH, 1 mmol/L Na_2_EDTA) to allow DNA denaturing. The electrophoresis was carried out for 15 min at 25 V and 300 mA. At the end of the electrophoresis, the slides were washed for 10 min with neutralization buffer (40 mmol/L Tris-HCl, pH 7.4), stained with 50 μL of 20 μg/mL ethidium bromide and observed under a light microscope equipped with a Leica DMCS epi-fluorescence system (stimulation wave length, 515–560 nm, emission wave length 590 nm) (Leica Microsystems, Germany) and with a Leica DC-100 video camera (Leica Microsystems, Germany). The samples from the comet assay were analyzed through a public domain image analysis system (NIH-image program), developed by the US National Institutes of Health and available on the Internet (http://rsb.info.nin.gov/nih-image) [30]. The length and moment of the tail were recorded for 50 images per sample.

### 2.6. Determination of Free Radicals Through a Lipid Peroxidation Assay

Free radical measurement was performed according to the Oxford Biomedical Research Inc. 2001 protocols [31]. This bioassay is based on the principle of a chromogenic reaction of *N*-methyl-2-phenylindole (MPI) with malonedialdehyde (MDA) or 4-hydroxyalkenals at 45 °C, which forms a stable chromophore that can be detected by spectrophotometry at a 586 nm absorbance.

Initially, blood was removed from the tissue by immersion in a cold isotonic saline solution; then the tissue was weighed and homogenized in 0.02 M phosphate buffer, pH 7.4 (20/30% w/v). To prevent sample oxidation, 10 μL of 0.5 M butylated hydroxytoluene were added per each mL of homogenized tissue. Coarse tissue particles were removed by centrifugation (3,000 g for 10 min at 4 °C). Total protein levels were measured in a sample aliquot by the Bradford method [32] and the samples were immediately frozen at −70 °C until the assay was carried out.

A volume of 650 μL of 10.3 nM MPI in a 1:3 mix of acetonitrile and methanol were added to 200 μL of sample in a microcentrifuge tube. The sample was smoothly mixed in a vortex and 150 μL of methanesulfonic acid were added, the mix was incubated at 45 °C for 1 h. Those samples showing sediment were centrifuged (15,000 g for 10 min). The supernatant was obtained and analyzed in a spectrophotometer at 586 nm.

### 2.7. Statistical Analysis

Data statistical analysis was carried out using the SPSS software. Values represent the means ± SEM. One-way ANOVA and Tukey’s multiple comparison tests were used to estimate the significance of the differences found among the groups. P *<* 0.05 was considered statistically significant.

## 3. Results

### 3.1 Micronuclei in Cells of Oral Mucosa Smears

**Figure 1.**
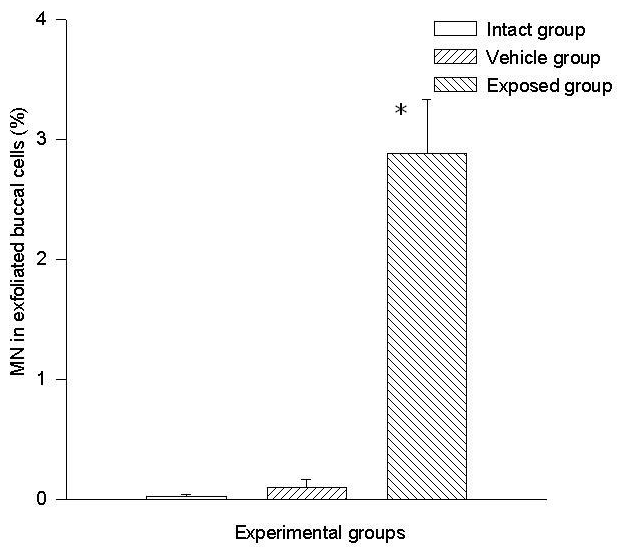
DDT effects on micronucleated cells frequency in buccal smears. Values are expressed as mean ± SEM. * = Significantly different from control groups at p < 0.001.

A statistically significant difference was evident when comparing the data of the exposed group (2.8 ± 0.44%) vs those of the vehicle control group (0.009 ± 0.006%) and those of the intact control group (0.002 ± 0.002). No significant differences were found when comparing data of the intact control group vs those of the vehicle control group (Figure 1).

### 3.2. Lymphocyte Single Cell Electrophoresis Test

**Figure 2.**
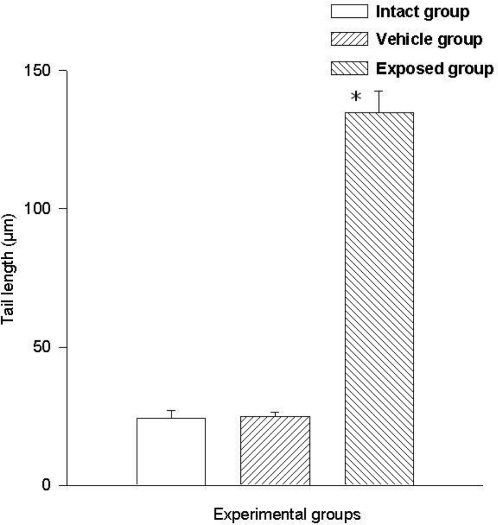
DDT effects on DNA migration in lymphocytes. Values are expressed as mean ± SEM. * = Significantly different from control groups at p < 0.001.

**Figure 3.**
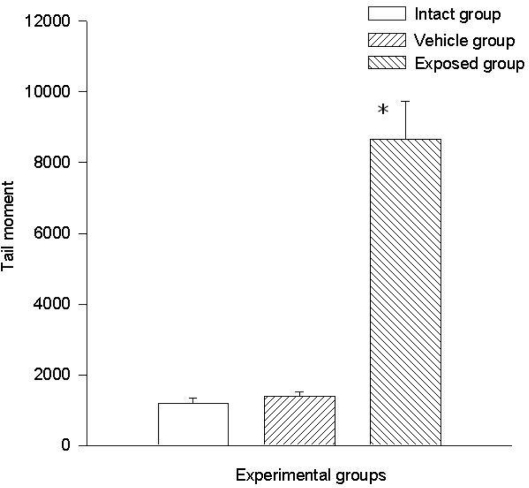
DDT effects on tail moment in lymphocytes. Values are expressed as mean ± SEM. * = Significantly different from control groups at p < 0.001.

Two parameters were taken into account in the comet test to evaluate the genotoxic effect of DDT: The comet’s tail length (μm) and the comet’s tail moment. Results demonstrated the existence of damage in the lymphocytes of exposed animals compared to those of the control groups. A statistically significant difference was observed when data from the exposed group was compared to those of the intact control and the vehicle control groups. This difference was noticed in both parameters. The average value difference between members of the exposed and the intact control group in tail length was 110.5 ± 6.7 μm (p < 0.001); the difference between the exposed and the vehicle control group was 110.08 ± 6.7 μm (p < 0.001). On the other hand no difference between values of the intact control group and those of the vehicle control group was observed. As for the comet tail moment, the difference of mean values between the exposed and the intact group was 7,463.15 ± 898.5 (p < 0.001); between the exposed and the vehicle control groups, the difference was 7,284.1 ± 898.5 (p < 0.001). When comparing the tail momentum values of the intact control group and those of the vehicle control group, no statistically significant difference was observed. Figures 2 and 3 show the mean values ± SEM for each group and for both parameters independently.

### 3.3. Single Cell Electrophoresis in Mammary Epithelial Cells

Comet tail length (μm) and comet tail moment were the two parameters that were considered to evaluate the genotoxic effect of DDT by means of the comet assay in mammary epithelial cells. The results obtained in mammary cells proved that there is a statistically significant difference when comparing the values of the exposed group with those of the intact control and the vehicle control groups, and this difference was noticed in both parameters.

**Figure 4.**
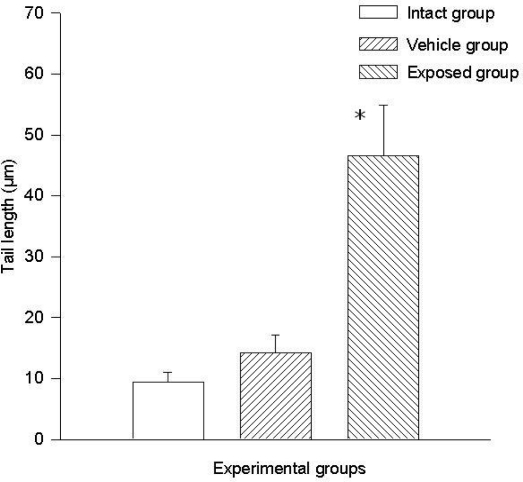
DDT effects on DNA migration in epithelial mammary cells. Values are expressed as mean ± SEM. * = Significantly different from control groups at p < 0.01.

**Figure 5.**
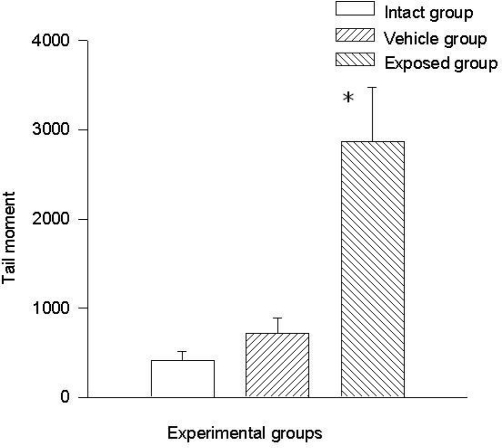
DDT effects on tail moment in epithelial mammary cells. Values are expressed as mean ± SEM. * = Significantly different from control groups at p < 0.01.

The difference of the means between the exposed group and the intact control group in tail length was 37.20 ± 7.28 μm (p < 0.01) and the difference between measurements of the exposed group against those of the vehicle control group was 32.30 ± 7.28 μm (p < 0.01). The difference between the values of the intact control group and those of the vehicle control group was 4.90 ± 7.28 μm. Regarding comet tail momentum, the differences between the values of the exposed group and those of the intact control group was 2,452.6 ± 523.8 (p < 0.01), and between the values of the exposed group and those of the vehicle control group was a 2,152.5 ± 523.85 (p < 0.01) difference. When comparing the values of the intact control group with those of the vehicle control group, the difference was 300.03 ± 523.8. Figures 4 and 5 show the mean values ± SEM for each group and for both evaluated parameters.

### 3.5. Free Radical Measurement

**Figure 6.**
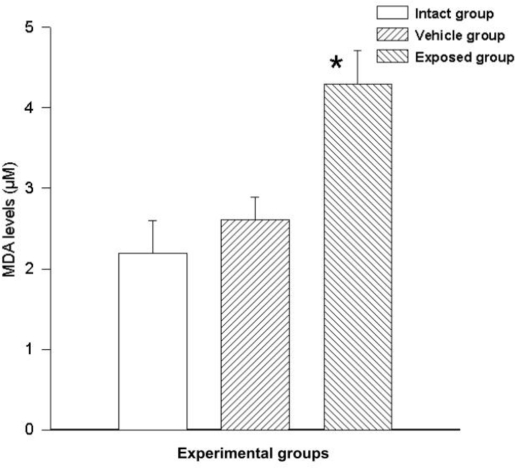
DDT effects on lipid peroxidation in epithelial mammary cells. Values are expressed as mean ± SEM. * = Significantly different from control groups at p < 0.05.

Free radicals were measured by MDA synthesis in the tissue; a statistically significant difference was observed between the measurements of the exposed group and those of the vehicle control group. The difference of the mean values from these groups was 1.68 ± 0.53 nmol/ml (p < 0.05). A similarly significant difference was found when comparing the values from the exposed group with those of the intact control group, where the value variation between these two groups was 2.10 ± 0.53 nmol/ml (p < 0.05). No significant differences were seen when comparing the data from the vehicle control group with those of the intact control group (0.42 ± 0.53) (Figure 6).

## 4. Discussion

Xenohormones can have a bi-functional behavior, depending on their mechanism of action, either through a genetic or hormonal route; this depends on their structure, concentration and exposure period [4]. On the other hand, it has been reported that exposure to some xenoestrogens and to estrogenic metabolites promotes the production of free radicals through the hydrogen peroxide (H_2_O_2_) reduction cycle, by means of the P_450_ oxidase and reductase enzymes, giving origin to the production of oxygen reactive species which might damage the genetic material [33]. The cause of the increase in oxidative damage may be through an xenoestrogenic mechanism or a genotoxic mechanism. Many other chemical carcinogens, such as benzo[a]pyrene and aflatoxin B1, are known to induce oxidative damage into DNA, which plays an important role on carcinogenesis [34], and the genotoxic effect on oral mucosa cells is demonstrated in the experimental models by MN formation after a long-term inhaled DDT exposure. Other authors have reported an increment in MN, chromosome aberration (CA) and sister chromatid exchange (SCE) in human lymphocytes exposed to complex mixtures of pesticides [35,36]. In this regard, our results are in agreement with those obtained by other research groups where the authors evaluated the induction of MN *in vitro* by exposing whale skin fibroblasts to organochlorine compounds, concluding that the exposure to high concentrations of chlordane, DDT or toxofane induced MN formation [37].

Regarding genotoxicity studies, those assays focused on the cytogenetic effects or breaking and loss of chromosomes have received special attention, since these effects are related to the development of various pathologies due to environmental contaminants, including cancer [38]. Depending on experimental conditions, various types of genetic damage such as: the breaking of DNA single or double strands, oxidative damage and alkali labile sites can be detected through the comet assay [39,40].

In the present work, the comet assay was performed to evaluate the DDT genotoxic effect due to a chronic exposure on lymphocytes and mammary epithelial cells. The results showed that DDT induces genetic damage in the studied cell tissues. The considered parameters used to determine genetic damage were the comet’s tail length and the tail’s moment because these parameters are the most frequently used to reflect the test sensitivity [41]. According to other authors, olive tail moment and tail length in the comet assay have shown to be the most useful parameters for assessing DNA damage [41,42].

Some authors have evaluated the genetic damage in leukocytes obtained from women suffering breast cancer, who were exposed to DDT and to some other environmental contaminants, finding no significant correlation with DDT exposure [42]. They comment that it could be explained in terms of the low blood DDT concentrations detected in those women.

In terms of the obtained data from mammary tissue, the results showed the same tendency as for lymphocytes, since a significant difference was noticed when comparing the exposed group data with that from the two control groups. As mentioned before, there are studies demonstrating that exposure to DDT damages the genetic material, and that the comet assay can be used to detect DNA damage due to environmental contaminants, as in the case of DDT [38,40]. In an experimental study carried out to evaluate the damage caused by different chemical compounds (DDT among them) in different organs from rats and mice, by means of the comet assay, Sekihashi *et al.* demonstrated that DDT induced damage in all organs except for the brain and the spinal cord [43]. Villarini *et al.* intended to associate DDT levels to DNA fragmentation in blood cells of indigenous women exposed to DDT, finding a large amount of comet images in their blood mononuclear cells [41].

Women suffering from breast cancer, showing mutations on the BRCA1 and BRCA2 genes, apparently are more susceptible to present genetic damage in others tissues than healthy individuals [44]. However, it is important to know whether aromatic hydrocarbons increase the risk for genetic damage or mutation in these genes in patients with breast cancer.

Exposure to environmental contaminants can lead to a biochemical unbalance or an adaptive response in living organisms [45]. In some cases, depending on the type and concentration of the toxic agent, its biotransformation can involve redox cycling reactions and/or uncoupled electron transference; these reactions are able to yield different reactive oxygen species derived from the chemical agent in question [46]. Those species may react with nucleic acids, lipids, carbohydrates and proteins, damaging the cell [47]. The generation of oxygen radicals and other pro-oxidant processes, have been linked to the etiology of several human diseases including cancer [48].

Lipid peroxidation is an identified cell damage mechanism in plants and animals, and it is used as an indicator of oxidative stress in cells and tissues. Lipid peroxides are unstable and break up into diverse complex forms; peroxides from polyunsaturated fatty acids produce malondialdehyde and 4-hydroxyalkenals, which are used as lipid peroxidation indicators [49,50]. The current study evaluates the influence of DDT over the free radicals production in mammary tissue in order to establish whether the induction of cellular damage is caused by oxidative stress. The results showed a significant increment in the lipid peroxidation rate in mammary cell membranes in DDT exposed rats in comparison to animals from both control groups. It has been reported that due to their high persistence in the environment and their ability to accumulate in the adipose tissue, polyhalogen cyclic hydrocarbons, some organophosphate pesticides and chlorinated herbicides, produce toxic alterations [51], mainly related to lipid peroxidation processes, in which reactive oxygen species are involved. High serum MDA levels and an increment in MDA-DNA adducts formation in mammary tissue have been reported in women suffering from breast cancer [52,53]. Chronic exposure to aromatic hydrocarbons, including DDT, have shown to produce lipid peroxidation in different animal tissues, including human cells [17,54]. In the current work, adult rats chronically exposed to DDT showed high a lipid peroxidation rate in their mammary tissue, reflecting an oxidative stress condition. It is a well known fact that oxidative stress plays a very important role in the carcinogenesis process; also, some facts indicate that reactive oxygen species are involved in cancer early stages and in its progression [55,56].

## 5. Conclusions

In summary, a genotoxic effect related to chronic exposure to environmental DDT in adult rats was revealed by means of an MN test and a comet assay. Two of the most accepted biomarkers used to detect DNA fragmentation and chromosome damage, phenomena that are directly related to cancer in various tissues. Moreover, the results related to oxidative stress further support that chronic exposure to environmental DDT generates tissue damage. Other systematic studies are needed in order to evaluate the association between environmental aromatic hydrocarbon exposure and the incidence of human disease.

## Abbreviations

DDT: 1,1,1-trichloro-2,2-bis-(chlorophenyl)-ethane;
MN: micronuclei;
MDA: malondialdehyde;
DDE: dichlorodiphenyldichloroethylene
